# Guillain–Barré Syndrome Associated With Zika Virus Infection: A Prospective Case Series From Mexico

**DOI:** 10.3389/fneur.2019.00435

**Published:** 2019-04-30

**Authors:** José Luis Soto-Hernández, Samuel Ponce de León Rosales, Edwin Steven Vargas Cañas, Graciela Cárdenas, Karina Carrillo Loza, José Alberto Díaz-Quiñonez, Irma López-Martínez, María-Eugenia Jiménez-Corona, Cuitláhuac Ruiz-Matus, Pablo Kuri Morales

**Affiliations:** ^1^Department of Infectious Diseases National Institute of Neurology and Neurosurgery Manuel Velasco Suárez, Mexico City, Mexico; ^2^Programa Universitario de Investigación en Salud UNAM, Mexico City, Mexico; ^3^Neuromuscular Clinic, Department of Neurology, National Institute of Neurology and Neurosurgery Manuel Velasco Suárez, Mexico City, Mexico; ^4^Department of Neurology, National Institute of Neurology and Neurosurgery Manuel Velasco Suárez, Mexico City, Mexico; ^5^Instituto de Diagnóstico y Referencia Epidemiológicos “Dr. Manuel Martínez Báez”, Mexico City, Mexico; ^6^División de Estudios de Postgrado, Facultad de Medicina, Universidad Nacional Autónoma de México, Mexico City, Mexico; ^7^Dirección General de Epidemiología, Mexico City, Mexico

**Keywords:** Zika virus, Guillain–Barré Syndrome, cranial neuropathies multiple, RT-PCR, flavivirus infection

## Abstract

**Background:** On May 2016, anticipating the rainy season from June to October in Mexico, we expected an increase in cases of Zika virus (ZIKV) infections. With the goal of identifying cases of GBS associated with ZIKV infection, a prospective joint study was conducted by a reference center for neurological patients and the Secretary of Health in Mexico City from July 2016 to November 2016.

**Methods:** Serum, cerebrospinal fluid, urine, and saliva were tested by RT-PCR for ZIKV, dengue virus, and chikungunya virus in patients referred from states with reported transmissions of ZIKV infection, and with clinical symptoms of GBS according to the Brighton Collaboration criteria. Clinical, electrophysiological, and long-term disability data were collected.

**Results:** In the year 2016 twenty-eight patients with GBS were diagnosed at our institute. In five hospitalized patients with GBS, RT-PCR was positive to ZIKV in any collected specimen. Dengue and chikungunya RT-PCR results were negative. All five patients had areflexic flaccid weakness, and cranial nerves affected in three. Electrophysiological patterns were demyelinating in two patients and axonal in three. Three patients were discharged improved in 10 days or less, and two patients required intensive care unit admission, and completely recovered during follow-up.

**Conclusion:** Our results are similar to those reported from the state of Veracruz, Mexico, in which out of 33 samples of urine of patients with GBS two had a positive RT-PCR for ZIKV. Simultaneous processing of serum, CSF, urine, and saliva by RT-PCR may increase the success of diagnosis of GBS associated to ZIKV.

## Introduction

In April 2016, a report of Epidemiological Surveillance for Zika virus (ZIKV) disease in Mexico reported 93 autochthonous laboratory-confirmed cases, collected between November 2015 and February 2016, and distributed amongst eight states of the country (1). In these patients, clinical manifestations were fever (96.6%), rash (93.3%), non-purulent conjunctivitis (88.8%), headache (85.4%), and myalgia (84.3%). No neurological manifestations were found among them. In 2015–2016, ZIKV infected individuals in South America, first in Northeast Brazil, rapidly disseminating to other South American, Caribbean and Central America countries, and reached North America by the end of 2016, with a parallel increase in cases of Guillain–Barré Syndrome (GBS) (2). Neurological disorders associated to ZIKV disease include now GBS, meningoencephalitis, myelitis, ophthalmic manifestations, and acute transient polyneuritis (3).

## Methods

A joint study was implemented between the National Institute of Neurology and Neurosurgery of Mexico City, and the Epidemiology Department of the Ministry of Health, with the aim of prospectively identifying ZIKV infection in new patients with GBS. We included patients coming from states with ongoing ZIKV transmission, diagnosed with GBS according to the Brighton criteria (4), from July to November 2016. The study was approved by the Ethics and Research Committees of both institutions, and written informed consent was obtained from all the patients included in accordance with the Declaration of Helsinki. We excluded patients with a positive human immunodeficiency virus serology, vaccinated patients in the last 6 months, and exposed to toxins. Upon admission, we applied the official assessment for case reporting recommended by Guideline of surveillance of ZIKV disease and its complications proposed by Pan American Health Organization and World Health Organization 2016 (5). The patients were examined by a neurologist who recorded demographic and clinical data of earlier systemic infection(s), neurologic symptoms, and signs and cerebrospinal fluid (CFS) data. Electrophysiological studies: Nerve conduction velocity studies were done in all patients using standard techniques, performed by a board-certified electrophysiologist and reviewed by two neuromuscular neurologists. The criteria to classify the results were as previously described (4, 6). Serum, CSF, saliva and urine samples of patients with GBS coming from states with ZIKV infection documented were collected within 24 h of admission, and processed by the Mexico National Reference Laboratory (Institute for Epidemiological Diagnosis and Reference). QIA amp Viral RNA Mini Kit (Qiagen, Hilden, Germany) was used for viral RNA isolation and the Superscript III RT-PCR system (Invitrogen, Carlsbad, CA, USA) and primers and probes as previously reported (7), for amplification for RNA fragments. A 760-bp fragment was used for partial characterization of the viral NS5 coding gene of ZIKV. Specimens were considered positive if target amplification was detected within 39 threshold cycles. With interest in detect possible simultaneous co-infections by dengue virus or chikungunya virus, all patient samples were tested with RT-PCR for the four serotypes of dengue virus and chikungunya virus according to previously described methods (8, 9). Patients were followed daily during hospitalization. Functional status (Hughes scoring system) (10) at hospital discharge and during follow-up was measured. We reviewed the records of discharge diagnoses of the neurology service in the years 2013–2015 to establish the annual median of cases of GBS for the last 3 years.

## Results

The diagnosis of GBS was established in 28 patients in our center during 2016. The median number of annual cases of GBS during the previous 3 years was 35, we did not observe an increase in the number of cases of GBS in the year of study, comparing with the previous years. In the study period (July to November 2016), six patients met the criteria of suspected case of ZIKV associated to GBS and five among them, met the criteria for confirmed case of ZIKV associated to GBS (5), Table 1 shows the characteristics of the patients. Only one patient (A) presented the classic picture of ZIKV symptoms, and all tested specimens in her, were positive by RT-PCR to ZIKV. In other three patients, non-specific viral symptoms were observed, and one patient did not have any systemic symptoms before the neurological illness. The median time from systemic symptoms to onset of neurological features was 4 days (IQR 3–7) Specimens for ZIKV RT-PCR test were obtained after a median of 9 days from the onset of the first symptom (IQR 7–12.5), it depended on the hospitalization date, since all samples were collected within 24 h. The NCS showed demyelinating pattern in two patients and axonal in three (see Table S1 in Supplementary Material). After admission, the duration of stay in the hospital was 10 days or less for three patients, that improved fast. Two patients required intensive care unit (ICU) admission, both had comorbidities and required tracheostomy and a gastrostomy. Patient B had a cranial trauma sequel 16 years earlier. He was admitted with quadriparesis and lower cranial nerves affected, remained for 5 days in the ICU, and he was discharged after 34 days in the hospital; during outpatient follow-up, he recovered his swallowing ability at 5 months, and demonstrated normal extraocular movements at 8 months. Our patient D was referred to us from another hospital with ventilator-associated pneumonia. Remained in the ICU for 22 days and was discharged after 52 days of hospitalization. Two months later, he was able to walk five meters without support (Hughes GBS score: 2) (10). On follow-up, 4 months later, swallowing and speech were fully reestablished. Four patients received immunomodulator treatment. No simultaneous co-infections with dengue or chikungunya virus by RT-PCR tests were found.

**Table 1:** Features of patients with Guillain Barré syndrome from México, July-November 2016.

**Clinical/laboratory data**	**Patient, Age range in years**				
	***A*, 35–39**	***B*, 25–29**	***C*, 35–39**	***D*, 75–79**	***E*, 60–65**
State of residence	Guerrero	Michoacán	Estado de México	Guerrero	Guerrero
Initial viral symptoms	Fever, headache, myalgias, polyarthralgia, retroocular pain, conjunctivitis, diarrhea, nausea, vomiting,	Headache, fever	Rash, pruritus, abdominal pain, diarrhea	None	Diarrhea
Time from systemic symptoms to neurological symptoms onset days	7	9	3	0	3
Neurologic symptoms/ signs	Distal paresthesias, upper and lower limbs weakness, bifacial palsy, oculomotor nerve palsy, generalized areflexia.	Upper and lower limbs weakness, bifacial palsy, dysphagia, dysarthria, generalized areflexia and dysautonomia.	Asymmetric upper and lower limbs weakness, allodynia, generalized areflexia and hypotonia.	Upper and lower limbs weakness generalized areflexia, hypotonia, oculomotor nerve palsy and bifacial palsy.	Distal paresthesias, upper and lower limbs weakness, generalized areflexia and hypotonia.
Days in hospital	6	34	6	52	10
Co-morbidities	None	Cranial trauma history	None	Obesity and smoking	None
Complications	None	ICU admission tracheostomy and gastrostomy	None	Pneumonia, ICU admission, tracheostomy and gastrostomy	None
Time from initial symptoms to CFS analysis (Days)	13	8	4	7	7
CSF					
Cell count x 10^6^ cells/mm3 Protein mg/dL	1 89	0 38	2 41	30 116	0 35
Glucose mg/dL	53	81	84	87	107
Positive PCR for ZIKV in/ Viral load, Threshold cycle Ct <39 is positive	Serum/ 36.18, CSF/ 31.08, urine/ 32.16, saliva/ 33.43	Saliva/ 33.61	Serum/ 35.52	Serum/ 38.62, urine/ 36.14	CSF/ 37.63, urine/ 38.11
Time from initial neurological symptoms to electrophysiological study (Days)	8	12	5	9	12
Electrophysiological patterns	AIDP Normal motor and sensory amplitudes, distal latency,conduction velocities and F waves.	AMAN Normal motor and sensory amplitudes, distal latency, conduction velocities and F waves.^†^	AMSAN Marked reduction of motor and sensory amplitudes, normal conduction velocities with slight prolonged latencies.	AMSAN Marked reduction of motor and sensory amplitudes, normal conduction velocities with slight prolonged latencies.	AIDP Marked prolonged distal latencies, reduced motor amplitude and absence of sensory potentials amplitudes, F waves absences. Slight reduction of conduction velocities.
Brighton criteria	2	2	2	1	2
Treatment	None, spontaneous improvement in 6 days	Plasma exchange (5 sessions)	IVIG 2 gr/kg/ 5 days	IVIG 2 gr/kg/ 5 days	Plasma exchange (4 sessions)
Hughes scale initial/at follow-up	2/1	5/1(9 months)	4/2 (2 months)	5/1 (8 months)	4/4 (at discharge)

† *Control electrophysiological study (8 months later) showed better median, cubital and tibial motors amplitudes*.

*AMAN, acute motor axonal neuropathy; AIDP, acute inflammatory demyelinating polyneuropathy; AMSAN, acute motor and sensory axonal neuropathy; ICU, intensive care unit*.

## Discussion

### Clinical Presentation and Course

The systemic symptoms that preceded the onset of GBS in our patients compared with those of other studies of GBS associated with ZIKV from Latin America and Caribbean, show that the absence of symptoms before neurological manifestation was observed in a study from Colombia in two of 42 subjects, and also it was noted that the systemic symptoms of ZIKV and the neurologic symptoms overlapped in another two patients (11). In a study from Brazil, in three of 27 patients having GBS associated with ZIKV, no viral prodrome preceded the neurological symptoms (12). A study from Puerto Rico found an acute antecedent illness to neurological symptoms in 57 of 71 patients (13). In Table 2, we present the number of cases with cranial neuropathy, of the largest published series of ZIKV associated GBS from Latin America and Puerto Rico (11–13). The number of patients with uni and bifacial palsy, ranged from 44 to 62%; the involvement of the bulbar cranial nerves or dysphagia and dysarthria occurred more frequently in the studies from Colombia and Puerto Rico (11, 13) than in the study from Brazil (12). In the report from Puerto Rico, at 6 months after neurologic illness onset, 53.5% of the patients reported abnormal tearing and 17.9% had difficulty drinking from a cup (13). A high number of patients were admitted to ICUs, with the percentage of such in the three above-mentioned series ranging from 33 to 66%. Not all studies reported the number of patients who were discharged from the hospital with tracheostomy or gastrostomy; however, these data highlight the important morbidity and threatening character for life of GBS associated with ZIKV infection. The duration of the neurological disease and the speed of recovery varies, with some patients showing a rapid recovery without treatment. In a study from Suriname (14) two out of three patients with GBS associated with Zika virus infection improved without treatment, one after an 11-day hospital stay, with a full recovery at 12 weeks, while other patient was discharged on Day 18, able to walk a small distance with assistance (14). On the other end, in the study from Puerto Rico, one-half of the study subjects were transferred to rehabilitation centers or skilled nursing facilities at the time of hospital discharge (13).

**Table 2:** Comparison of data from Latin American and Puerto Rico studies of GBS associated with Zika virus infection.

**Country/ number of patients included/ (reference)**	**Number of samples tested by RT-PCR/ number positive (%)**	**Median time from viral symptoms to testing/ (IQR or range)**	**Cranial neuropathy**	**ICU admission/tracheostomy/ gastrostomy**
Colombia/68/(10)	Serum 31/1 (3.2) CSF 30/3 (10) Urine 24/16 (66.6)	16.5/(IQR: 11.5–19.7)	Bifacial palsy 34; bulbar cranial nerves 15; cranial nerves III, IV, and VI 7	40/NA/NA
Brazil/40^*^/(11)	Serum 40/3 (7.5) CSF 40/2 (5)	6/(4–7)	Facial palsy 11; bifacial palsy 5; dysphagia 3	9/NA/NA
Puerto Rico/71/(12)	Serum 99/23 (23.2) CSF 31/1 (3.2) Urine 43/5 (11.6) Saliva 18/0 (0)	9/(1–92)	Facial weakness 44; dysphagia 38; dysarthria 27	47/6/5
Mexico/5/present report	Serum 5/3 CSF 5/2 Urine 5/3 Saliva 5/2	9/(IQR: 7.0–12.5)	Bifacial palsy 3; cranial nerves III, IV, and VI 3; cranial nerves IX and X in one	2/2/2

* *27 had GBS, five had encephalitis, two had transverse myelitis, and one had chronic inflammatory demyelinating polyneuropathy. ICU, intensive care unit; NA, not available*.

A recent study, described a ZIKV infection-associated acute transient polyneuritis (3), they were three patients with a short viral prodrome of 24–48 h, and clinical findings consistent with a mild, self-limited, distal, sensorimotor neuropathy, with unrevealing electrophysiologic and CSF testing, nevertheless the patients had positive ZIKV RT-PCR in CSF and blood in two and blood and urine in one, all their neurologic symptoms resolved in 7–10 days. Since symptoms and imaging findings tracked closely with ZIKV viremia, the authors hypothesize that there may be a direct neuropathic effect of ZIKV leading to inflammation and nerve swelling. We underline that our patient A with high viral load to ZIKV in all specimens, and albuminocytological dissociation, recovered from the motor deficit and affected cranial nerves in 7 days. It is necessary to study more similar cases, to define whether direct neural damage due to ZIKV itself could have a role in the pathogenesis of these clinical forms. In México, a study reported by del Carpio-Orantes et al. (15), presented the results of 34 hospitalized patients with GBS from the state of Veracruz, in year 2016 out of 18 urine samples, ZIKV RT-PCR was positive in two patients; also, two patients had positive IGM Dengue antibodies in serum. In the year 2017 they studied 16 patients with GBS, none of them had RT-PCR positive for ZIKV. Interestingly in the same 16 patients they carried out detection in feces of Campylobacter by PCR and found 12 (75%) positive, which suggests that in that region of Mexico, Campylobacter is a most frequent cause of GBS than the ZIKV.

### RT-PCR Testing

With regard to RT-PCR testing, in our five patients, simultaneous samples taken at hospital admission of serum, CSF, urine, and saliva produced 10 positive results. Table 2 summarizes the results of RT-PCR tests of three large studies of GBS associated with ZIKV, the positive RT-PCR percentage vary, as also the times of obtaining the samples after the onset of viral symptoms. ZIKV RT-PCR was positive in serum with a range from 3.2 to 23.2% and, for CSF, from 3.2 to 10%. For urine, the positive values were higher, from 11.6 to 66.6%. Finally, for saliva, the study from Puerto Rico (13) reported that none of 18 samples were positive, while we found two positive RT-PCR results from five samples in the present study. Considering that, in Latin America and the Caribbean, in some areas, general hospitals with neurologists and ICUs may be scarce, and therefore lumbar punctures may not be practiced, it seems advisable that in cases with GBS in which the association with ZIKV is suspected, testing serum, urine, and saliva simultaneously may increase the possibility of confirming the diagnosis until more specific serologic tests for antibodies are introduced and made widely available.

### Limitations

This small case series has several limitations. It is a study from a single center, with a reference bias, since we included patients from surrounding states, able to present for evaluation at emergency room via their own resources. We include patients from states of México with transmission of ZIKV, which indicated that transmitting vectors were present. The altitude of Mexico City (2250 meters above sea level) where most of our hospitalized GBS cases come from, is considered by CDC as minimal likelihood for mosquito-borne ZIKV transmission (16), and only one of the six patients tested was a Mexico City resident with travel history to a state with ZIKV transmission, and his RT-PCR studies were negative. We did not test for antibodies against viruses by cross-reactivity, and we could not study other possible infectious causes of GBS, and also, the study period of 5 months was short, all due to economic restrictions.

## Conclusion

We conclude as other Latin American and Caribbean studies that GBS associated with ZIKV infection presents in a significant number of cases with cranial neuropathy; up to two-thirds of affected patients may require admission to ICUs; the number of cases confirmed by RT-PCR in serum or CSF is low; and urine and saliva tests show variable results or are not done in some studies. The simultaneous processing of serum, urine, and saliva by RT-PCR may increase the success of diagnosis in areas without access to lumbar punctures, and when possible, other infectious agents should be sought even in areas endemic to arboviruses. Patients suffering from GBS due to ZIKV require an early evaluation in centers with qualified neurologists and an ICU stay for optimal care.

## Ethics Statement

This study was approved by the ethics and research committees of the Directorate of the Secretary of Health in Mexico City and the National Institute of Neurology and Neurosurgery Manuel Velasco Suárez, México City, Mexico. All participants signed informed consent prior to participation in this study.

## Author Contributions

JS-H, SP, EV, CR-M, GC, KC, JD-Q, IL-M, M-EJ-C, and PK designed the study protocol and wrote the manuscript. JS-H, EV, GC, and KC cared for the patients and collected the data. JD-Q, IL-M, M-EJ-C, CR-M, and PK were responsible for the molecular diagnostic tests. All authors reviewed and approved the final version of the manuscript.

### Conflict of Interest Statement

The authors declare that the research was conducted in the absence of any commercial or financial relationships that could be construed as a potential conflict of interest.
