# Supplementary material for: Guillain–Barré Syndrome Associated With Zika Virus Infection: A Prospective Case Series From Mexico
Source: Front Neurol. 2019 Apr 30;10:435. doi: 10.3389/fneur.2019.00435 (PMC6502985; doi:10.3389/fneur.2019.00435)
Supplement: Supplementary file 1 [file Table_1.docx]

**Table S1. Neuroconduction characteristics in confirmed cases of Zika-Virus associated to GBS from Mexico 2016.**

| **Patient** | **A** | **B**  **Initial** | **B**  **Control after 8 months** | **C** | **D** | **E** |
| --- | --- | --- | --- | --- | --- | --- |
| Motor neuroconduction DL ms (CV m/s) Amp mV  Median nerve  Ulnar nerve  Tibial nerve  Peroneal nerve | 2.9(52)11.25  2.4(66)8.48  4.5(49)13.3  3.9(51)6.56 | 2.8(62)7.86  2.2(65)10.82  3.5(49)16.15  4.4(53)5.31 | 4.1(59)10.95  3.2(63)11.68  4.9(45)10.81  5.2(44)7.11 | NR  NR  6.8(49)0.07  7 (62) 0.56 | NR  3.7(70)0.37  NR  NR | 6.1(42)0.69  4.8(47)5.42  NR  NR |
| Sensitive neuroconduction  DL ms (CV m/s) Amp uV  Median nerve  Ulnar nerve  Sural nerve | 2.4(59)49.15  2.5(56)26.75  2.4(58)33.28 | 2.6(54)23.3  2.7(52)7.45  2.5(56)18.5 | 2.5(56)80.94  2.9(48)39.69  3.5(40)30.40 | NR  NR  NR | 2.5(56)7.77  2.8(51)4.61  NR | NR  NR  NR |

F waves were present in patients A and B, and absent in the patients C, D and E. Amp: Amplitude, CV: conduction velocity, DL: distal latency. ms: millisecond, m/s: meter/second, mV: millivolt. uV: microvolt. NR: non register.
